# Assessment of absolute risk of life-threatening cardiac events in long QT syndrome patients

**DOI:** 10.3389/fcvm.2022.988951

**Published:** 2022-10-07

**Authors:** Meng Wang, Derick R. Peterson, Eleonora Pagan, Vincenzo Bagnardi, Andrea Mazzanti, Scott McNitt, David Q. Rich, Christopher L. Seplaki, Valentina Kutyifa, Bronislava Polonsky, Alon Barsheshet, Deni Kukavica, Spencer Rosero, Ilan Goldenberg, Silvia Priori, Wojciech Zareba

**Affiliations:** ^1^Division of Cardiology, Clinical Cardiovascular Research Center, University of Rochester Medical Center, Rochester, NY, United States; ^2^Division of Epidemiology, Department of Public Health Sciences, University of Rochester Medical Center, Rochester, NY, United States; ^3^Department of Biostatistics and Computational Biology, University of Rochester Medical Center, Rochester, NY, United States; ^4^Department of Statistics and Quantitative Methods, University of Milan-Bicocca, Milan, Italy; ^5^Molecular Cardiology, Istituti Clinici Scientifici Maugeri, Istituto di Ricovero e Cura a Carattere Scientifico, Pavia, Italy; ^6^Department of Molecular Medicine, University of Pavia, Pavia, Italy; ^7^Division of Pulmonary and Critical Care, Department of Medicine, University of Rochester Medical Center, Rochester, NY, United States; ^8^Department of Environmental Medicine, University of Rochester Medical Center, Rochester, NY, United States; ^9^Cardiology Division, Rabin Medical Center, Petah Tikva, Israel; ^10^Sackler Faculty of Medicine, Tel Aviv University, Tel Aviv, Israel; ^11^Division of Cardiology, University of Rochester Medical Center, Rochester, NY, United States; ^12^Molecular Cardiology, Fundación Centro Nacional de Investigaciones Cardiovasculares, Madrid, Spain

**Keywords:** long QT syndrome, risk prediction, sex, syncope, beta blocker, cardiac arrest, implantable cardioverter defibrillator

## Abstract

**Background:**

Risk stratification in long QT syndrome (LQTS) patients is important for optimizing patient care and informing clinical decision making. We developed a risk prediction algorithm with prediction of 5-year absolute risk of the first life-threatening arrhythmic event [defined as aborted cardiac arrest, sudden cardiac death, or appropriate implantable cardioverter defibrillator (ICD) shock] in LQTS patients, accounting for individual risk factors and their changes over time.

**Methods:**

Rochester-based LQTS Registry included the phenotypic cohort consisting of 1,509 LQTS patients with a QTc ≥ 470 ms, and the genotypic cohort including 1,288 patients with single LQT1, LQT2, or LQT3 mutation. We developed two separate risk prediction models which included pre-specified time-dependent covariates of beta-blocker use, syncope (never, syncope while off beta blockers, and syncope while on beta blockers), and sex by age < and ≥13 years, baseline QTc, and genotype (for the genotypic cohort only). Follow-up started from enrollment in the registry and was censored at patients’ 50s birthday, date of death due to reasons other than sudden cardiac death, or last contact, whichever occurred first. The predictive models were externally validated in an independent cohort of 1,481 LQTS patients from Pavia, Italy.

**Results:**

In Rochester dataset, there were 77 endpoints in the phenotypic cohort during a median follow-up of 9.0 years, and 47 endpoints in the genotypic cohort during a median follow-up of 9.8 years. The time-dependent extension of Harrell’s generalized C-statistics for the phenotypic model and genotypic model were 0.784 (95% CI: 0.740–0.827) and 0.785 (95% CI: 0.721–0.849), respectively, in the Rochester cohort. The C-statistics obtained from external validation in the Pavia cohort were 0.700 (95% CI: 0.610–0.790) and 0.711 (95% CI: 0.631–0.792) for the two models, respectively. Based on the above models, an online risk calculator estimating a 5-year risk of life-threatening arrhythmic events was developed.

**Conclusion:**

This study developed two risk prediction algorithms for phenotype and genotype positive LQTS patients separately. The estimated 5-year absolute risk can be used to quantify a LQTS patient’s risk of developing life-threatening arrhythmic events and thus assisting in clinical decision making regarding prophylactic ICD therapy.

## Introduction

Congenital long QT syndrome (LQTS) is a genetic channelopathy manifested by QT prolongation on the electrocardiogram and an increased risk of ventricular tachyarrhythmia and sudden cardiac death (SCD) (1, 2). It is a major cause of SCD in young subjects without structural heart disease (3). To date, disease-causing mutations have been identified in 17 genes (4), with three genes (KCNQ1, KCNH2, and SCN5A) accounting for over 90% of genotype-positive patients (5), Risk stratification in LQTS patients is important for optimizing patient care and clinical decision regarding treatment, especially the decision to implant implantable cardioverter defibrillators (ICD) in young patients with lifetime risk of complications (6, 7). Previous studies have identified several risk factors including prolonged heart rate corrected QT interval (QTc, (7–13) history of syncope, (8–10, 13, 14) male sex before adolescence, (9, 12, 15, 16) female sex after adolescence, (9, 12, 15, 16) protective beta-blocker treatment, (12, 17) and genotype (1, 7, 13). However, there is a need for a risk stratification algorithm able to integrate all individual risk factors and predict a patient’s absolute risk of developing a life-threatening arrhythmic event in a given time window. A recent study by Mazzanti et al. (4) developed an algorithm for LQTS patients with calculations of absolute risk of cardiac events based on QTc and genotype.

We developed a risk prediction algorithm with prediction of 5-year absolute risk of life-threatening arrhythmic events [aborted cardiac arrest, sudden cardiac death (SCD), or appropriate ICD shocks] in LQTS patients, accounting for individual risk factors (age, sex, QTc, syncope, beta-blocker use) and their changes over time, using data from the Rochester LQTS Registry. At first, an assessment of the risk can be based on the abovementioned clinical variables since genetic testing results (if test performed) usually are available several weeks later. When genetic test results become available, they could be used to further refine estimation of individual risk. Given that genotype data were not available for about one-third of patients in the Registry and some patients with prolonged QTc were tested negative, we developed two algorithms for two cohorts separately: (1) a clinical algorithm that only included clinical risk factors (QTc, age, sex, history of syncope, use of beta-blockers) for patients with QTc prolongation, regardless of genotype or with unknown yet genotype (*phenotypic cohort*); (2) and a genetic algorithm that included all abovementioned clinical risk factors plus genotype for LQTS genotype positive patients, regardless of a patient’s QTc duration (*genotypic cohort*). This manuscript describes development of these models and subsequent external validation in a large LQTS registry cohort followed in Pavia, Italy, and proposes an on-line calculator estimating the risk of life-threatening cardiac events in LQTS patients.

## Materials and methods

### Study population

LQTS patients included in the study were from the Rochester LQTS Registry (18), which is the US portion of the International LQTS Registry established in 1979 (18), Participants in the Registry are LQTS patients and their family members enrolled across the United States. Patients included in the present study were enrolled before December 21, 2016. The phenotypic cohort included 1,509 patients with a QTc ≥ 470 ms, regardless of their genotype (i.e., these patients could be genotype positive or negative), and the genotypic cohort included 1,288 patients with a single mutation in KCNQ1 (LQT1), KCNH2 (LQT2), or SCN5A (LQT3) genes and whose QTc was available (regardless of the specific value of QTc). There were 698 patients who had both QTc ≥ 470 and a single mutation in KCNQ1, KCNH2, or SCN5A that were included in both cohorts. Patients who developed life-threatening arrhythmias (definition provided below) before registry enrollment were excluded from the study. All participants provided written informed consent, and the study protocol was approved by the Institutional Review Board at the University of Rochester Medical Center.

### Follow-up and assessment of the endpoint

Participants in the Registry were followed annually using mailed questionnaires since enrollment. For probands, annual follow-up with patients’ physicians was also performed. The endpoint was the first occurrence of a life-threatening arrhythmic event, defined as a composite endpoint of aborted cardiac arrest, SCD, or appropriate ICD shock. Aborted cardiac arrest was defined as abrupt onset of loss of consciousness that requires external defibrillation as part of the resuscitation (8). It was assessed by patients’ self-report at enrollment and at each annual follow-up and verified by medical records whenever possible. Mortality was assessed by contact with relatives of the deceased using available medical documentation. SCD was defined as death abrupt in onset without evident cause if witnessed or death that was not explained by any other cause if it occurred in a non-witnessed setting such as sleep (8). SCDs were adjudicated by LQTS investigators based on a description of the circumstances around the time of death and medical records when available. Appropriate ICD shocks were defined as shocks delivered for torsade de pointes or polymorphic ventricular tachycardia or ventricular fibrillation. The type of arrhythmia that triggered a shock and the appropriateness of the shock were ascertained by an event adjudication committee based on all available information including reports from the patient’s electrophysiologist.

### Assessment of clinical risk factors and genotype

A 12-lead electrocardiogram (ECG) was obtained at Registry enrollment for all subjects included in the study. ECGs were read centrally by the study physicians at the University of Rochester Medical Center. QTc was calculated using Bazett’s formula. For probands, QTc was measured from the first ECG received and read by the reading center showing a qualifying QTc of >440 ms. For family members, QTc was measured from the earliest ECG available. History of syncope and beta-blocker use were self-reported by the study subject at enrollment and at each annual follow-up. In the presence of a self-reported arrhythmic event, the patient’s physician was contacted to verify the information. Genetic testing results reported to the Registry came from research or commercial laboratories. A patient may have undergone comprehensive testing for all known LQTS genes or just targeted mutation-specific genes. The registry only documented genetic testing results that had been reported by genetic testing laboratories or patient’s physicians. Assessments of these clinical risk factors and genotype used in risk prediction were performed without knowledge of a patient’s outcome status since they were assessed prior to outcome occurrence.

### Statistical analysis

#### Multivariable models

We used time-dependent Cox models, including pre-specified LQTS prognostic factors, to estimate the 5-year absolute risk of developing a life-threatening arrhythmic event. Robust sandwich estimates of standard errors were used to account for clustering of events within the same family. Follow-up started at enrollment and was censored on the date of subjects’ 50th birthday, date of death due to reasons other than SCD, or date of last registry contact, whichever occurred first.

Pre-specified factors in the model for the phenotypic cohort included QTc, age at enrollment, an interaction term between sex and time-dependent age < and ≥13 years (to account for the sex risk-reversal occurring around the time of adolescence), time-dependent beta-blocker use (yes vs. no), and time-dependent history of syncope (no syncope, syncope that occurred while off beta-blockers, and syncope that occurred while on beta-blockers). For the genotypic cohort, in addition to these clinical factors, genotype (LQT1, LQT2, and LQT3) was also included in the model. We further examined interactions among genotype, age, and sex; no significant interactions were found.

We checked the assumption of linear functional form for QTc using cumulative martingale residuals and no significant violations were found in either cohort. Therefore, QTc was analyzed as a linear term. When checking the assumption of linear functional form for age at enrollment using the same approach, violations were found. Therefore, we categorized age into five groups with 10 years as interval length (i.e., 0– < 10, 10– < 20, 20– < 30, 30– < 40 and 40–50 years). Given that the effect estimates for age groups 20– < 30, 30– < 40 and 40–50 years compared to 0– < 10 years were similar in both cohorts, and there is a biological basis of human development (childhood, adolescence, and adulthood), we combined the age groups of 20– < 30, 30– < 40 and 40–50 years. Thus, age at enrollment was analyzed as a three-level categorical variable (i.e., 0– < 10 years, 10– < 20 years, and 20–50 years) in the final models for both cohorts. The three groups of time-dependent history of syncope were mutually exclusive at any given time point, with “no syncope” as the reference group. If a patient had both histories of syncope on beta-blockers and syncope off beta-blockers, he/she was classified in the group of syncope on beta blockers (i.e., the group expected to have a higher risk). Only syncopal events that occurred prior to ICD implantation were counted. Proportional hazard assumptions were tested via interactions between each risk factor and log (time), and no violations were found.

#### Estimation of 5-year risk

Assuming that the covariate pattern (and thus risk score z) will remain fixed for t years, the event-free survival from time 0 to time t for an individual patient can be predicted using the following equation, derived from the Cox model:


$$S\left(t|z=riskscore\right)=S_{0}{\left(t\right)}^{\exp\left(\mathrm{risk}\,\mathrm{score}-\mathrm{reference}\,\mathrm{risk}\,\mathrm{score}\right)},$$

where the risk score is the linear combination of all log hazard ratios (regression coefficients) relevant to the covariate pattern of the patient (i.e.,$\mathrm{risk}\,\mathrm{score}=\sum_{\text{i}=1}^{\text{p}}\beta_{\text{i}}\,{\text{x}}_{\text{i}}$, where β is the regression coefficient and x is the level for each risk factor). S_0_(t) is the cumulative event-free survival probability from time 0 (i.e., enrollment) to time t (e.g., *t* = 5 years) for an individual with the reference risk score, estimated via Breslow’s estimator of the baseline cumulative hazard function (19).

Since it is necessary to hypothesize some reasonable 5-year future covariate trajectory when forecasting risk using a time-dependent Cox model, we assumed that the covariate pattern will remain fixed for the 5-year prediction window since enrollment. Therefore, in most cases when predicting 5-year risk for a patient, only the baseline risk score (i.e., at enrollment) of that patient was used. However, one exception was to predict risk for female patients whose age at enrollment was between 8 and 13 years (i.e., 8 years < age at enrollment < 13 years). Given that the 5-year prediction window for these patients included 13 years, and based on the model the effect estimate for female vs. male before age 13 was different from that after age 13, it would not be appropriate to still assume a fixed risk score over the 5 years. The Supplementary material provide a detailed description of how to compute predicted risk when the 5-year prediction window for a female includes age 13. Briefly, we divided the 5-year prediction window into two intervals: starting age to year 13, and age 13 to ending age (starting age+5). We first computed the event-free survival probability using the risk score at the starting age for the first interval, then computed the event-free survival probability using the risk score at age 13 years (assuming the level of each risk factor remains the same as it is at the starting age) for the second interval.

#### Assessment of model performance

For each of the two models, we first assessed apparent model discrimination (i.e., discrimination performance estimated directly from the dataset that was also used to develop the prediction model) using time-dependent extension of Harrell’s generalized C-statistic proposed by Kremers (20). Furthermore, both models were externally validated in an Italian cohort of 1,481 individuals younger than 50 years at enrollment and carriers of a single mutation in one of the three major LQTS genes (KCNQ1, KCNH2, or SCN5A) who were followed-up prospectively at the Molecular Cardiology clinic of the IRCCS ICS Maugeri in Pavia, Italy. The model for the phenotypic cohort was validated in a subsample of 681 patients with QTc ≥ 470 ms, while the model for the genotypic cohort was validated in all 1,481 patients. For each model, a time-dependent risk score was calculated for each patient in the Italian cohort, using the parameters estimated from the training dataset (the Rochester cohort). The risk score was then included in a time-dependent Cox model as the only covariate. The C-statistic, calibration coefficient (i.e., beta coefficient of the risk score), and their 95% confidence intervals obtained from this model were reported. All analyses were performed using SAS software (version 9.4; SAS Institute Inc.).

## Results

### Clinical characteristics of long QT syndrome patients

Clinical characteristics of patients included in the Rochester phenotypic and genotypic cohorts are shown in Table 1. There were 698 patients included in both cohorts. Females accounted for 60.2% in the phenotypic cohort and 56.2% in the genotypic cohort. Average age at enrollment was 20 years in both cohorts. Prevalence of syncope at enrollment was higher in the phenotypic cohort compared to the genotypic cohort (39.5% vs. 29.7%). Prevalence of a history of beta blocker use (phenotypic vs. genotypic: 74.5% vs. 78.0%) and prevalence of a history of ICD implantation (21.0% vs. 21.1%) were similar between the two cohorts. In the phenotypic cohort, 49.8% patients were genotype positive, 9.5% were negative for the mutations tested, and 40.8% were either not tested or had missing data on genotype. In the genotypic cohort, 45.8% patients were mutation carriers with a QTc < 470 ms.

**TABLE 1:** Patients characteristics at enrollment in the Rochester phenotypic and genotypic cohort.

Characteristics	Phenotypic cohort (*N* = 1,509)	Genotypic cohort (*N* = 1,288)
**Females**	908 (60.2)	724 (56.2)
**Age at enrollment, years**		
<10	430 (28.5)	412 (32.0)
10– < 20	434 (28.8)	293 (22.7)
20– < 50	645 (42.7)	583 (45.3)
Mean age	20 ± 15	20 ± 16
**QTc. ms**		
<470	0 (0)	590 (45.8)
470– < 500	820 (54.3)	342 (26.6)
500– < 550	459 (30.4)	242 (18.8)
≥550	230 (15.2)	114 (8.9)
Mean QTc, ms	507 ± 44	477 ± 50
Heart rate, bpm	83 ± 26	82 ± 28
Age at ECG, year	18.7 ± 14.7	18.8 ± 15.6
History of syncope*		
No syncope	913 (60.5)	906 (70.3)
Syncope while off beta blockers	498 (33.0)	327 (25.4)
Syncope while on beta blockers	98 (6.5)	55 (4.3)
**History of treatment**		
Beta-blockers	1,124 (74.5)	1,005 (78.0)
Sodium channel blockers	72 (4.8)	46 (3.6)
Left cardiac sympathetic denervation	27 (1.8)	13 (1.0)
Pacemaker	146 (9.7)	82 (6.4)
ICD	317 (21.0)	272 (21.1)
**Genotype**		
LQT1 (single mutation)	334 (22.1)	582 (45.2)
LQT2 (single mutation)	291 (19.3)	549 (42.6)
LQT3 (single mutation)	73 (4.8)	157 (12.2)
Others (single mutation)	12 (0.8)	0 (0)
Multiple mutations	41 (2.7)	0 (0)
Negative for mutations tested	143 (9.5)	0 (0)
Not tested/unknown	615 (40.8)	0 (0)
**No. of life-threatening events**	77	47
SCD	30	14
ACA	14	7
Appropriate ICD shocks	33	26
**Follow-up: Total person-years**	15,505	14,221
Median (years)	9.0	9.8
Interquartile range (years)	4.4–15.1	5.3–15.9
**Incidence rate of the endpoint (No. of events/100 person-years)**	0.50	0.33

Data are mean ± SD or *N* (%). *If a patient experienced both syncope while on beta blockers and syncope while off beta blockers, the patient was classified in the group of syncope while on beta blockers (i.e., the group with a presumed higher risk).

### Endpoints during follow-up

In the Rochester phenotypic cohort, during a follow-up of 15,505 person-years (median: 9.0 years) 77 patients developed the endpoint (30 SCD, 14 aborted cardiac arrest, and 33 appropriate ICD shocks, Table 1). The incidence rate of the first life-threatening arrhythmic event was 0.5 per 100 person-years. In the Rochester genotypic cohort, during a follow-up of 14,221 person years (median: 9.8 years) 47 patients developed the endpoint (14 SCD, 7 aborted cardiac arrest, and 26 appropriate ICD shocks). The incidence rate of the first life-threatening arrhythmic event was 0.33 per 100 person-years.

### Prediction models

**FIGURE 1:**
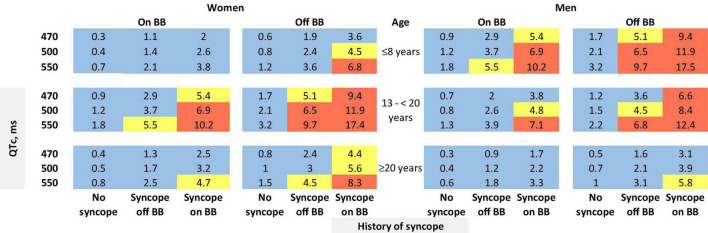
Predicted 5-year risk of life-threatening arrhythmic events (aborted cardiac arrest, sudden cardiac death, or appropriate ICD shocks) for some example risk factor combinations for patients with a QTc ≥ 470 ms. Blue color indicates a predicted 5-year risk of < 4%, yellow color indicates a predicted 5-year risk of 4– < 6%, and red color indicates a predicted 5-year risk of ≥6%. Numbers in this figure are percentage. BB: beta-blockers. We chose three QTc values (470, 500, and 550 ms) and three age groups at baseline (≤8 years, 13– < 20 years, and ≥20 years) as examples. We did not show the age group of 8–13 years. Given that the 5-year risk prediction interval includes age 13 for patients aged 8–13 years at baseline, in this age group each unique age has a unique predicted risk. Thus, graphical presentation can be cumbersome. The online risk calculator can compute predicted risk for any age points.

The risk prediction model for the phenotypic cohort is shown in Table 2. QTc and time-dependent history of syncope and beta-blocker use were all significantly associated with the risk of life-threatening arrhythmic events. Compared to patients who were younger than 10 years at enrollment, those aged 10– < 20 years had a non-significant 31% decrease in the risk (HR = 0.69, 95% CI: 0.38–1.25, *P* = 0.221), and those aged 20–50 years had a significant 69% decrease in the risk (HR = 0.31, 95% CI: 0.16–0.62, *P* = 0.001). Although not significant, before age 13 years, females had a 64% lower risk of life-threatening arrhythmic events than males (HR = 0.36, 95% CI: 0.11–1.22, *P* = 0.102), whereas from 13 years onward, females had a 45% higher risk than males (HR = 1.45, 95% CI: 0.84–2.50, *P* = 0.185). There was a significant interaction between sex and time-dependent age < 13 vs. ≥13 years (*P* = 0.038). Figure 1 shows the predicted 5-year risk since enrollment for some example covariate patterns.

**TABLE 2:** Model predicting ACA, SCD, or appropriate ICD shock after enrollment in 1,509 LQTS patients with QTc ≥ 470 ms (No. of events = 77, 30 SCD, 14 ACA, and 33 shock).

Variables in the model	β	HR	95% CI		*P*
**QTc**, per 10 ms increase	0.08	1.09	1.05	1.13	<0.001
**Age at enrollment** (**Ref: 0– < 10 years)**					
10– < 20 years	-0.37	0.69	0.38	1.25	0.221
20–50 years	-1.16	0.31	0.16	0.62	0.001
**Time-dependent syncope (Ref: No syncope)**					
Syncope while off BB	1.14	3.12	1.59	6.11	0.001
Syncope while on BB	1.77	5.87	2.89	11.90	<001
**Time-dependent beta-blocker (yes vs. no)**	-0.58	0.56	0.34	0.93	0.024
**Sex by time-dependent age (female vs. male)**					
<13 years	-1.01	0.36	0.11	1.22	0.102
≥13 years	0.37	1.45	0.84	2.50	0.185
C statistic	0.784 (0.740–0.827)				

Robust sandwich estimates of standard errors were used. *P*-value for interaction between time-dependent age and sex: 0.038.

**FIGURE 2:**
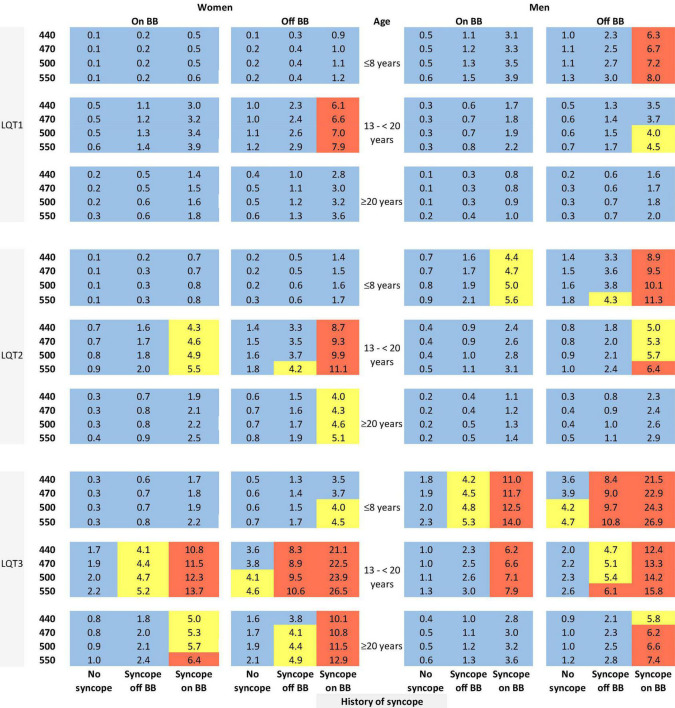
Predicted 5-year risk of life-threatening arrhythmic events (aborted cardiac arrest, sudden cardiac death, or appropriate ICD shocks) for some example risk factor combinations for patients with a single mutation in LQT1, LQT2, or LQT3. Blue color indicates a predicted 5-year risk of <4%, yellow color indicates a predicted 5-year risk of 4% to < 6%, and red color indicates a predicted 5-year risk of ≥ 6%. Numbers in this figure are percentage. BB, beta-blockers.

**FIGURE 3:**
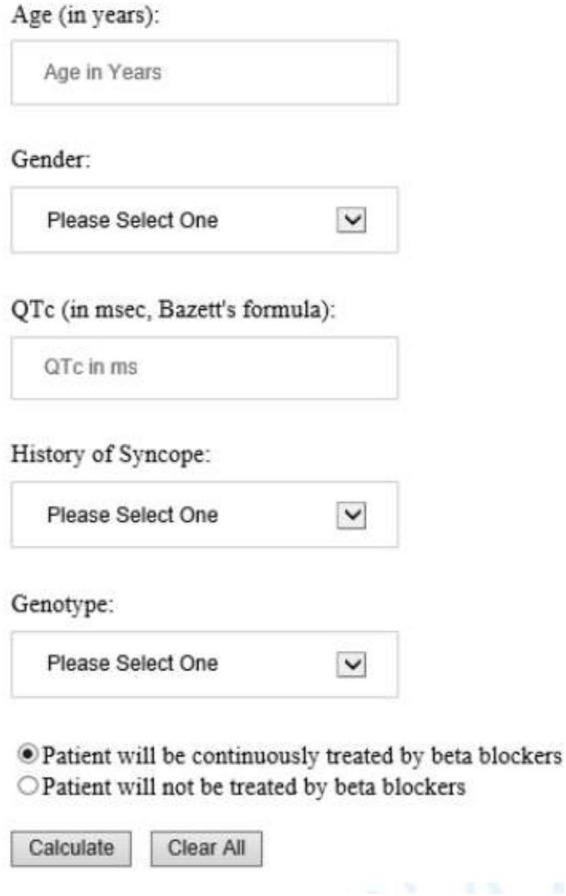
University of Rochester Long QT Syndrome Risk Calculator predicting 5-year risk of life-threatening arrhythmic events (aborted cardiac arrest, sudden cardiac death or appropriate ICD shock). The drop-down list of Gender included Male and Female; History of Syncope included Occurred while on BB, Occurred while off BB, and No syncope history. Genotype included LQT1, LQT2, LQT3, and Unknown/test negative.

The risk prediction model for the genotypic cohort is shown in Table 3. Similar to the model for the phenotypic cohort, time-dependent history of syncope and beta-blocker use were significantly associated with the risk of life-threatening arrhythmic events. However, the effect size of QTc was smaller and non-significant (HR = 1.02, 95%CI: 0.96–1.09, *P* = 0.442). In sensitivity analysis, QTc was analyzed using a piecewise linear spline with a knot at 470 ms to allow different slopes for the QTc- arrhythmia risk association in the two QTc ranges (<470 ms and ≥470 ms), but there was insufficient evidence of such nonlinearity (*p* = 0.885 and Schwartz’s Bayesian Information Criterion increased from 597.7 to 601.6). The pattern of association for age at enrollment and sex by time-dependent age (<13 vs. ≥13 years) was similar to that of the phenotypic model. Compared to LQT1 patients, LQT2 patients had a non-significant 44% higher risk of life-threatening arrhythmic events (HR = 1.44, 95% CI: 0.75–2.77, *P* = 0.279), and LQT3 patients had a significantly over threefold higher risk (HR = 3.75, 95% CI: 1.74–8.07, *P* = 0.001). Figure 2 shows the predicted 5-year risk since enrollment for some example covariate patterns. For both the phenotypic and genotypic cohorts, formulae with examples of risk calculations were given in Supplementary material. An online risk calculator is shown in Figure 3 and can be accessed via the following link: LQTS Risk Calculator^1^.

**TABLE 3:** Model predicting ACA, SCD, or appropriate ICD shock after enrollment in 1,288 LQTS patients with singe LQT1, LQT2, or LQT3 mutation (No. of events = 47, 14 SCD, 7 ACA, and 26 shock).

Variables in the model	β	HR	95% CI		*P*
**QTc**, per 10 ms increase	0.02	1.02	0.96	1.09	0.442
**Age at enrollment (Ref: 0–10 years)**					
10–20 years	-0.60	0.55	0.26	1.15	0.112
20–50 years	-1.40	0.25	0.11	0.56	0.001
**Time-dependent syncope (Ref: No syncope)**					
Syncope while off BB	0.87	2.38	1.10	5.16	0.028
Syncope while on BB	1.88	6.54	2.91	14.65	<001
**Time-dependent beta-blocker (yes vs. no)**	-0.73	0.48	0.25	0.91	0.025
**Sex by time-dependent age (female vs. male)**					
<13 years	-1.92	0.15	0.02	1.13	0.066
≥13 years	0.58	1.79	0.91	3.51	0.090
**Genotype (Ref: LQT1)**					
LQT2	0.36	1.44	0.75	2.77	0.279
LQT3	1.32	3.75	1.74	8.07	0.001
C statistic	0.785 (0.721–0.849)				

Robust sandwich estimates of standard errors were used. *P*-value for interaction between time-dependent age and sex: 0.018.

### Validation of model performance

Patient characteristics of the genotypic cohort from Pavia, Italy, has been described previously showing similar clinical characteristics to Rochester genotypic cohort including 52% (vs. 56%) of females, mean QTc of 471 ± 45 ms (vs. 477 ± 50 ms), similar proportions of LQTS genotypes, follow-up duration and event rates of 0.47% (vs. 0.33%) events per year (4).

In the Italian cohort (*N* = 1,481, all patients had a single mutation in LQT1, LQT2, or LQT3), during a follow-up of 12,616 person-years 57 patients developed the endpoint with an incidence rate of 0.45 per 100 person-years. Of the 1,481 patients, 681 had a QTc ≥ 470 ms. During a follow-up of 6,193 person-years, 50 of the 681 patients developed the endpoint with an incidence rate of 0.81 per 100 person-years.

The time-dependent extension of Harrell’s generalized C-statistics for the phenotypic model and genotypic model were 0.784 (95% CI: 0.740–0.827, *p* < 0.001) and 0.785 (95% CI: 0.721–0.849, *p* < 0.001), respectively, in the Rochester cohort. The C-statistics obtained from external validation in the Italian cohort were 0.700 (95% CI: 0.610–0.790, *p* < 0.001) and 0.711 (95% CI: 0.631–0.792, *p* < 0.001) for the two models, respectively. The calibration coefficient (beta) obtained from external validation for the phenotypic model was 0.822 (95% CI: 0.513–1.131), with *p* < 0.001 for the null hypothesis of beta = 0 (implying significant discrimination) and a *p* = 0.259 for the null hypothesis of beta = 1 (insufficient evidence of miscalibration). The calibration coefficient for the genotypic model was 0.744 (95% CI: 0.491–0.997), with *p* < 0.001 (significant discrimination) and *p* = 0.048 (significant miscalibration) for the two hypotheses, respectively.

## Discussion

The risk prediction algorithms for life-threatening arrhythmic events in LQTS patients proposed by the present study integrate several risk factors including age, sex, QTc, history of syncope, beta-blocker treatment, and genotype. The algorithm for the phenotypic cohort could be applied to patients with unknown yet genotype or patients who test negative for currently known LQTS mutations. The algorithm that included genotype could be applied to patients with a single mutation in LQT1, LQT2, or LQT3. Both algorithms demonstrated good discrimination in external validation (C-statistics ≥0.70 with >97.5% confidence that C-statistics ≥0.61 and *p* < 0.001). These algorithms with prediction of 5-year absolute risk of life-threatening cardiac events including aborted cardiac arrest, sudden cardiac death or appropriate ICD shock may be used in clinical practice to help identify high-risk patients requiring ICD therapy.

Different from the traditional approach that only uses baseline data when developing a risk prediction model, we included time-dependent covariates, accounting for changes in risk factors (i.e., beta blockers and syncope) over time. We believe this approach more accurately quantified the effect of each risk factor on the risk of life-threatening arrhythmic events compared to approaches that only use covariate status at baseline. We proposed a novel yet simple and familiar looking method to predict individual survival functions based on the time-dependent Cox model. Our approach assumes that a patient’s risk profile will remain fixed over the prediction time interval (e.g., 5-years), while placing no restrictions or assumptions on the time-dependent covariate trajectory outside that interval (for details, see Supplementary Methods). When interpreting the predicted risk, this assumption of fixed risk profile should be considered. Once a patient’s risk profile changes, the patient needs to be re-evaluated. The risk stratification method used by current clinical practice guidelines for LQTS is based on a crude combination of individual risk factors, primarily QTc and the presence or absence of symptoms (21), failing to account for the difference in the strength of associations between each individual factor and the risk of life-threatening arrhythmic events. By contrast, the algorithm developed by the present study integrates individual risk factors by assigning each factor a weight of its effect size, and it is able to quantify a patient’s absolute risk. Furthermore, the algorithm can estimate risk for both patients under beta-blocker treatment and patients who cannot take beta-blockers due to intolerance or contraindication. Multivariable risk assessment avoids overlooking patients with multiple marginal risk factors and over-treating patients with only one isolated risk factor such as syncope history (22). Our risk stratification algorithm suggests that some patients without a history of syncope may have a higher risk of life-threatening arrhythmic events than patients with a history of syncope. For example, a LQT3 boy younger than 8 years with a QTc of 550 ms who never experienced syncope and will be continuously treated by beta blockers has a predicted 5-year risk of 2.3%, which is higher than the 5-year predicted risk for a LQT1 male adult with the same QTc (i.e., 550 ms) and a history of syncope while on beta blockers who will be continuously treated by beta blockers (predicted risk = 1.0%). However, it should be noted that syncopal events in LQTS patients may be due to reasons other than arrhythmias such as vasovagal syncope and orthostatic hypotension, and the Registry data were not able to differentiate different types of syncope. This may also be the case in real world clinical settings when the exact reason for a syncopal event cannot be determined (e.g., a patient came to see a physician after experiencing a syncopal event). The nature of syncope and the likelihood that it is arrhythmic should be carefully evaluated and considered when estimating a patient’s risk and making treatment decisions.

The approach presented in the manuscript reflects real-world scenario when patients suspected for or diagnosed with LQTS are evaluated first without genetic testing available. The risk stratification approach proposed in this manuscript allows physicians to use existing clinical information without knowledge of genetic results at first. When genetic testing becomes available, the risk stratification model allows the use of risk calculator that accounts for genetic results. Furthermore, the risk could be re-evaluated at different age or at the time when new clinical information is available (i.e., new syncopal episode on beta-blocker).

A recent study by Mazzanti et al. also developed a risk stratification algorithm with calculations of 5-year absolute risk of life-threatening arrhythmic events in LQTS patients with a QTc >460 ms while not taking beta-blockers (4). However, the algorithm only considered two risk factors QTc and genotype. In our study, we included QTc, age at enrollment, sex by time-dependent age, and time-dependent history of syncope and beta-blocker use in the phenotypic model and additionally LQTS genotype in the genotypic model. Both models demonstrated good discrimination as suggested by an external validation C-statistic of ≥0.70. For the phenotypic model, the calibration coefficient (0.822, 95% CI: 0.513–1.131) was not significantly different from 1 (*P* = 0.259), thus providing insufficient evidence of miscalibration. However, for the genotypic model, the calibration coefficient (0.744, 95% CI: 0.491–0.997) was significantly different from 1 (*P* = 0.048), indicating evidence of miscalibration. Nevertheless, given that the point estimate of the calibration coefficient was as large as 0.744 and different from 0 (*p* < 0.001), the risk score was still capable of discrimination in the validation cohort and the magnitude of miscalibration was not large.

It is worth noting that the aim of this risk prediction tool is to provide objective data on prognosis to facilitate the clinical decision-making of treatment, especially ICD implantation, rather than to simply categorize patients into high or low risk groups using arbitrary thresholds. As already noted by others (23), there is no universal consensus on the level of absolute risk that justifies ICD therapy. The risk of life-threatening arrhythmic events should be interpreted as a continuum. When making decisions regarding ICD implantation for the primary prevention of life-threatening events, patients and physicians need to weigh the risks and benefits of the device in the context of the patient’s clinical condition and preference. Nevertheless, current guidelines for other cardiac diseases have made recommendations regarding the risk cut-off that warrants ICD implantation. For example, based on the 2014 European Society of Cardiology Guidelines on Diagnosis and Management for Hypertrophic Cardiomyopathy, a 5-year risk of ≥6% could be used as the risk category for ICD recommendation (24).

The long-term prospective follow-up (median follow-up was 9.0 and 9.8 years for the phenotypic and genotypic cohort, respectively) was a major strength of the present study. By using enrollment as baseline, recall bias was minimized and the study population was more representative of concurrent patients under medical care. Additionally, we proposed a novel method to predict absolute risk based on time-dependent Cox models, which enabled us to include time-dependent syncope and beta blockers in our risk prediction models and thus account for changes in risk factors over time. However, several limitations of our study should be considered when interpreting our findings. First, the majority of our study population were females (60.2% in the phenotypic cohort and 56.2% in the genotypic cohort). It is known that the risk pattern of life-threatening arrhythmic events between males and females throughout their lifetimes is very different, and females have their distinct risk factors such as the post-partum high risk period (25), probably due to the influence of sex hormones (25–29). Although it would be ideal to build different models for males and females separately, our sample was not sufficiently large to perform this sex-specific analysis. Second, Caucasian non-Hispanic white (93.8% in the phenotypic cohorts and 96.7% in the genotypic cohorts) predominated in our cohorts. Therefore, our findings may not be generalizable to patients of other racial and ethnic origins. Third, to avoid informative censoring and to account for the fact that patients were still at risk of life-threatening arrhythmic events after ICD implantation, we included appropriate ICD shocks in the composite endpoint. However, appropriate ICD shock is not a perfect surrogate of life-threatening arrhythmic events. Data on detailed ICD programming (e.g., programmed delay of ICD therapy) were not available for every patient. Thus, we were not able to perform analyses focusing on prolonged ventricular tachycardia or fibrillation. In fact, given that it is impossible to know what would happen to a patient who experienced an appropriate ICD shock had the shock not been delivered, there is no way to ascertain life-threatening arrhythmic events with absolute accuracy after ICD implantation, even if details about ICD programming are available. Some syncopal events may be due to non-arrhythmic causes and our study was not able to differentiate arrhythmic vs. non-arrhythmic syncope. However, given the strong effect estimates for syncope variables in our models, most syncopal events documented in the registry should be arrhythmic. In future studies, the incremental predictive value of provocation testing and more detailed genetic information such as mutation locations and functions of genetic variants as well as pathogenicity should be evaluated in larger combined cohorts.

## Conclusion

This study proposed two risk stratification algorithms of the first life-threatening arrhythmic event, separately for patients with unknown and known genotype. Model performance was satisfying in an external cohort for both algorithms. Although genetic testing is increasingly used, it is not easily accessible in many parts of the world and initially physicians need to evaluate risk while waiting for genetic test results. We integrated individual risk factors including age, sex, QTc, history of syncope, beta-blocker treatment, and genotype (for genotype positive patients only). The estimated 5-year absolute risk can be used to quantify a patient’s risk and thus assist clinicians in decision making regarding prophylactic ICD therapy.

## Data availability statement

The datasets presented in this article are not readily available because the datasets are restricted to investigators and collaborating groups. Requests to access the datasets should be directed to WZ, wojciech.zareba@heart.rochester.edu.

## Ethics statement

The studies involving human participants were reviewed and approved by the Research Subject Review Board, University of Rochester Medical Center, Rochester, NY. Written informed consent to participate in this study was provided by the participants or the participants’ legal guardian/next of kin.

## Author contributions

MW, DP, DR, CS, VK, IG, and WZ contributed to the concept and design of the study. MW drafted the manuscript. MW, DP, SM, BP, and WZ contributed to acquisition, analysis, or interpretation of data. MW and EP performed statistical analyses. All authors contributed to critical revision of the manuscript for important intellectual content.

## Footnotes

1
https://www.urmc.rochester.edu/clinical-cardiovascular-research/lqts-registry/lqts-registry.aspx
