# Supplementary material for: Assessment of absolute risk of life-threatening cardiac events in long QT syndrome patients
Source: Front Cardiovasc Med. 2022 Oct 7;9:988951. doi: 10.3389/fcvm.2022.988951 (PMC9585302; doi:10.3389/fcvm.2022.988951)
Supplement: Supplementary file 1 [file Data_Sheet_1.pdf]

Supplementary materials

Supplemental Methods

To predict 5-year risk for a female patient whose age at enrollment was between 8 and 13 years, the 5-year prediction window needs to be divided into two intervals: enrollment to <13 years and 13 years to 5 years post enrollment. The 5-year risk can then be computed using the following formula:

$$1 - \mathrm{S}(t_1|z_0) * \frac{\mathrm{S}(t_2|z_1)}{\mathrm{S}(t_1|z_1)}$$

where z_0 denotes the patient's risk score at enrollment, $\mathrm{S}(t_1|z_0)$ denotes the cumulative event-free survival probability from enrollment to age 13 (i.e. t_1=13-age at enrollment) if the risk score were to remain fixed at z_0 from enrollment to age 13, z_1 denotes the new risk score accounting for change in time-dependent age (from before to after age 13) and thus in the effect estimate of female (all other risk factors remained the same as they were at baseline), $\mathrm{S}(t_2|z_1)$ denotes the cumulative event-free survival probability from enrollment to 5 years post enrollment (i.e. t_2= 5 years), if the risk score were to remain fixed at z_1 from enrollment to 5 years post enrollment, and $\mathrm{S}(t_1|z_1)$ denotes the cumulative event-free survival probability from enrollment to age 13, if the risk score were to remain fixed at z_1 from enrollment to age 13.

As can be inferred from the above notations, $\frac{\mathrm{s}(t_2|z_1)}{\mathrm{s}(t_1|z_1)}$ is the conditional survival (conditional on event-free survival to age 13 years) from age 13 to 5 years post enrollment. Given that by using this formula, any evolution of the patient's covariate pattern from time 0 to time t_1 (e.g. from enrollment to age 13) has been completely conditioned away, this predicted conditional survival function depends only on the portion of the baseline hazard from time t_1 to time t_2 (i.e.

from age 13 years to 5 years post enrollment) and on the covariate pattern trajectory from time t_1 to time t_2.

Examples of calculating predicted 5-year risk

The cumulative event-free survival from time 0 (i.e. enrollment) to time t for an individual with any given risk score (assuming that the risk score will remain fixed from time 0 to time t) can be calculated using the equation:

$$S(t|z = risk\ score) = S_0(t)^{\exp(\sum_{i=1}^{p} \beta_i x_i - reference\ risk\ score)},$$

where $S_0(t)$ is the survival function (cumulative event-free survival probability from time 0 to time t) for a patient with the reference risk score. In the examples below, the reference patient (risk score=0) in the phenotypic cohort was defined as a male patient with a QTc of 470ms, age at enrollment < 10 years, no syncope history, and off beta blockers. The reference patient in the genotypic cohort was defined as a male LQT1 patient with a QTc of 470ms, age at enrollment < 10 years, no syncope history, and off beta blockers.

Example 1: A 20-year-old male patient with a QTc = 550ms and a history of syncope while on beta blockers. Assuming that his risk factors will not change and he will be continuously treated by beta blockers, his 5-year risk of a life-threatening arrhythmic event since age 20y can be calculated as

$$\hat{P}_{endpoint\ at\ 5y\ since\ age\ 20y} = 1 - S(5y|z)$$

$$= 1 - 0.983232^{\exp\left(0.08246*[\frac{550-470}{10}]-1.16373+1.76929-0.57985\right)}$$

$$= 1 - 0.983232^{\exp(0.68539)} = 3.3\%,$$

where 0.983232 is the 5- year event-free survival from time 0 (i.e. enrollment) for a reference patient.

Example 2: A 10-year-old girl with a QTc of 510 ms and a single LQT2 mutation. She has no syncope history. Assuming that her risk factors will not change and she will be continuously treated by beta blockers, her 5-year risk of a life-threatening arrhythmic event since age 10 can be calculated using the following formulae:

$$S(3y|z_0) = 0.994282^{\exp\left(0.02349*\left[\frac{510-470}{10}\right]-0.60304-0.73255\ -1.91856+0.36233\right)}$$

$$= 0.994282^{\exp(-2.79786\ )}$$

$$S(3y|z_1) = 0.994282^{\exp\left(0.02349*\left[\frac{510-470}{10}\right]-0.60304-0.73255+0.58295+0.36233\right)}$$

$$= 0.994282^{\exp(-0.29635\ )}$$

$$S(5y|z_1) = 0.989449^{\exp\left(0.02349*\left[\frac{510-470}{10}\right]-0.60304-0.73255+0.58295+0.36233\right)}$$

$$= 0.989449^{\exp(-0.29635)}$$

$$\hat{P}_{\ endpoint\ at\ 5y\ since\ age\ 10y} = 1 - S(3y|z_0) \times \frac{S(5y|z_1)}{S(3y|z_1)}$$

$$= \ 1 - 0.994282^{\exp(-2.79786\ )} \times \left(\frac{0.989449}{0.994282}\right)^{\exp(-0.29635)} = 0.4\%,$$

where Z_0 denotes the risk score at age 10 years and Z_1 denotes the risk score at age 13 years. As can be seen from the formula, the level of each risk factor remains the same as it is at age 10. The only change is the beta for female (changed from −1.91856 to 0.58295 from < 13 years to ≥ 13 years). Baseline survival probabilities 0.994282 and 0.989449 are the cumulative event-free survival probabilities from time 0 (i.e. enrollment, age 10) to age 13 years and age 15 years, respectively, for a reference patient.

We identified 236 mutations from 352 families in our genotypic cohort. Twelve of the 352 families had more than one mutation (all individual patients had single mutation). Table S1-S3 provide a list of these identified mutations, along with the number of families and individual patients carrying each mutation. Number of penetrance was defined as the number of individual carriers with a QTc ≥ 470ms, and percentage of penetrance was defined as the percentage of individual carriers with a QTc ≥ 470ms.

	Table S1. List of mutations identified in the genotypic cohort-LQT1				
Mutation	Number of families	Number of patients	Number of patients with events	#penetrance	%penetrance
448 insG	1	3	0	1	33%
639+5G>A	1	3	0	2	67%
A150fs/133	1	4	0	2	50%
A178P	1	5	0	2	40%
A178T	1	1	0	1	100%
A302E	1	1	0	1	100%
A302V	1	4	0	0	0%
A341E	2	11	0	5	45%
A341V	2	4	0	4	100%
A344/sp	1	1	0	1	100%
A344A/sp	3	24	1	16	67%
A344V	1	1	0	1	100%
A46T	1	6	0	1	17%
A525T	1	3	0	1	33%
A590T	1	2	0	0	0%
D242Y	1	2	0	1	50%
D317G	1	3	1	3	100%
E160K	1	8	3	5	63%
E284K	1	2	0	2	100%
F340del	1	1	0	1	100%
G168R	9	68	0	40	59%
G269D	1	20	0	9	45%
G269S	5	25	0	14	56%
G292D	1	1	0	0	0%
G314S	3	6	1	3	50%
G325R	3	5	0	4	80%
G568R	3	9	1	7	78%

G57V	1	1	0	0	0%
H455Y	1	2	0	1	50%
I567S	1	2	0	1	50%
I567T	3	6	0	4	67%
IVS1+3G>C	1	3	0	1	33%
IVS10-1 G>T	1	5	0	1	20%
IVS2+5 G>A	3	5	0	2	40%
K557E	1	2	0	2	100%
K598K	1	12	2	4	33%
L191fs+90X	1	2	0	2	100%
L191fs/90	3	6	0	2	33%
L250P	1	1	0	1	100%
L266P	7	23	1	19	83%
L273F	6	17	0	12	71%
L353P	1	3	0	2	67%
M1V	1	1	0	0	0%
M520R	1	2	0	1	50%
P343S	1	1	0	1	100%
P400fs/62	1	4	0	3	75%
Q530X	2	12	0	6	50%
R174C	1	2	0	2	100%
R190Q	1	1	0	0	0%
R190W	1	4	0	1	25%
R195fs/40	1	3	0	1	33%
R243C	3	5	0	2	40%
R259C	1	7	1	2	29%
R259L	2	4	0	3	75%
R360G	1	3	0	3	100%
R366W	1	5	0	3	60%
R452Q	1	2	0	0	0%
R518Q	1	1	0	0	0%
R518X	7	15	0	7	47%
R539W	1	1	0	1	100%
R555C	2	8	0	6	75%
R562S	1	3	0	3	100%
R591H	2	10	0	7	70%
R594Q	3	7	0	3	43%
S225L	5	12	0	8	67%
S276del	1	1	0	1	100%
S277L	1	4	0	2	50%

S277del	1	2	0	0	0%
S349W	3	13	2	3	23%
S349X	1	8	0	2	25%
S389Y	1	1	0	1	100%
S546L	3	4	0	3	75%
S566F	2	2	1	2	100%
T312I	1	8	0	7	88%
V254M	6	67	1	47	70%
V307del sp	1	2	0	2	100%
V310I	1	2	0	0	0%
V524G	2	6	0	4	67%
V576I	1	1	0	1	100%
W188X	1	1	1	1	100%
W305C	1	2	0	2	100%
W305S	1	12	0	5	42%
Y171X	2	9	1	5	56%
Y281C	1	3	0	1	33%
Y315C	2	8	0	1	13%

	Table S2. List of mutations identified in the genotypic cohort-LQT2				
Mutations	Number of families	Number of patients	Number of patients with events	#penetrance	%penetrance
1157P fs+111X	1	9	0	3	33%
A561T	3	5	2	4	80%
A561V	5	12	1	9	75%
A614V	5	13	1	11	85%
A78P	1	2	0	0	0%
A83fs/37	1	1	0	1	100%
A913V	1	2	0	0	0%
C39R	1	2	0	0	0%
C44X	1	2	0	1	50%
C52AfsX8	1	1	0	0	0%
D102A	1	2	0	1	50%
D501G	1	3	0	1	33%
D501N	1	1	0	1	100%
D609N	1	5	0	4	80%
D774Y	2	2	0	2	100%
D793fs	1	2	0	1	50%
D837G	2	3	0	1	33%
D837Y	1	1	0	1	100%

E214X	1	6	0	3	50%
E788D	1	3	1	1	33%
E788K	1	4	0	1	25%
E900fsX973	1	1	0	1	100%
E90K	1	1	0	0	0%
E929fs	1	1	0	0	0%
F29L	1	6	1	2	33%
F627L	1	2	2	1	50%
F805C	1	6	0	6	100%
F805S	1	1	0	1	100%
G1006AfsX51	1	1	0	0	0%
G1036D	1	7	0	0	0%
G306W	1	2	0	1	50%
G47V	1	7	1	4	57%
G53R	1	6	0	5	83%
G584S	3	7	0	3	43%
G601S	1	4	0	0	0%
G604S	2	6	0	3	50%
G626fsX713	1	5	0	2	40%
G628S	1	1	0	1	100%
G648S	1	3	0	2	67%
G657S	1	1	1	1	100%
G749V	1	3	0	1	33%
G903R	1	2	0	0	0%
G921fsX973	2	3	0	1	33%
G925VfsX49	1	2	0	2	100%
G969VfsX5	1	4	0	1	25%
H309fs51X	1	7	0	4	57%
H70R	5	19	1	11	58%
H739HfsX63	1	1	0	0	0%
I31T	1	1	0	0	0%
I571L	2	6	0	2	33%
I571M	1	1	0	1	100%
I593R	1	4	0	3	75%
I711V	1	5	0	0	0%
K638E	1	3	1	3	100%
K638del	1	1	0	0	0%
L1066V	1	4	0	1	25%
L296CfsX64	1	4	0	3	75%
L380fsX433	1	8	0	4	50%
L552S	1	2	0	1	50%

L615F	1	3	0	3	100%
L799spl?	3	4	0	3	75%
L86R	1	8	0	7	88%
L987CfsX70	1	1	0	1	100%
M554fsX721	1	2	0	0	0%
M756V	1	2	0	2	100%
N33T	2	8	0	0	0%
N629I	1	1	0	1	100%
N629S	2	3	0	3	100%
N633S	1	4	1	4	100%
N861I	1	3	0	2	67%
N996I	1	6	0	6	100%
P1075L	1	1	0	1	100%
P151+179X	1	7	1	1	14%
P310fsX359	1	1	0	1	100%
P596L	1	2	0	2	100%
P632A	1	2	0	2	100%
P721L	1	2	0	2	100%
P72L	1	6	0	6	100%
P72Q	3	14	1	6	43%
P902QfsX71	1	2	0	2	100%
P926fsX940	2	2	0	2	100%
P968fs	1	2	1	1	50%
Q1046fs	1	1	0	0	0%
Q11X	1	2	0	2	100%
Q376spl?	4	16	0	3	19%
Q450X	1	3	0	3	100%
Q576SfsX18	1	1	0	0	0%
R1005fs+50X	1	1	0	1	100%
R1014X	3	8	0	5	63%
R1035G fsX22	1	1	0	0	0%
R1035fsX1056	2	5	0	3	60%
R148W	1	4	0	1	25%
R176PfsX156	1	1	0	1	100%
R176W	3	5	0	1	20%
R181fs/20	1	1	0	0	0%
R366X	2	6	0	3	50%
R534C	2	2	0	1	50%
R56Q	2	13	0	7	54%
R582C	1	1	0	0	0%
R685H	1	2	0	0	0%

R744X	1	1	0	1	100%
R752W	3	28	0	11	39%
R784Y	1	1	0	1	100%
R823W	2	10	0	7	70%
R954fs+X	1	1	0	1	100%
S182X	1	4	0	1	25%
S250X	1	1	0	0	0%
S26I	1	4	0	2	50%
S428X	1	1	0	1	100%
S818L	2	16	0	10	63%
S855R	1	4	0	0	0%
S937N	1	2	0	0	0%
T613M	4	4	0	4	100%
V377fs+9X	1	2	0	2	100%
V612L	1	1	0	1	100%
V822M	2	26	1	12	46%
W1001X	2	30	1	16	53%
W398X	1	1	0	1	100%
W410S	1	3	0	2	67%
W568fsX593	1	15	1	7	47%
W585X	1	1	0	1	100%
Y493H	1	1	0	1	100%
Y597C	1	2	0	2	100%

	Table S3. List of mutations identified in the genotypic cohort-LQT3				
Mutations	Number of families	Number of patients	Number of patients with events	#penetrance	%penetrance
A1088T	1	1	0	1	100%
A1330T	1	1	0	0	0%
E1781G	1	1	0	1	100%
E1784K	6	27	2	21	78%
F1596I	1	1	0	0	0%
F1617del	1	1	0	0	0%
G615E	1	3	0	1	33%
I1448L	1	1	0	0	0%
I1470T	1	1	0	1	100%
I1768V	1	20	1	7	35%
L1501V	1	2	0	1	50%
L1560F	1	2	0	0	0%

M1766V	1	8	2	2	25%
N1325S	1	10	1	3	30%
N406K	1	1	0	0	0%
P1021S	1	2	0	1	50%
P627L	1	1	0	0	0%
Q1507_P1509del	4	41	3	30	73%
Q692K	1	1	0	0	0%
R1623L	1	2	1	0	0%
S216L	1	6	0	0	0%
T1304M	2	6	0	0	0%
T1779M	1	4	0	1	25%
T370M	1	10	0	0	0%
V1667I	1	1	0	1	100%
V1777M	1	1	0	1	100%
Y1795C	1	1	1	1	100%
Y314H	1	1	0	0	0%
