# Supplementary material for: Assessment of absolute risk of life-threatening cardiac events in long QT syndrome patients
Source: Front Cardiovasc Med. 2022 Oct 7;9:988951. doi: 10.3389/fcvm.2022.988951 (PMC9585302; doi:10.3389/fcvm.2022.988951)
Supplement: Supplementary file 2 [file Data_Sheet_2.ZIP › Online Calculator New Formula/main.html]

Risk Calculator


# University of Rochester Long QT Syndrome Risk Calculator

Date of Birth:  
  
   
  
Gender:  

Please Select One
Male
Female
   
  
QTc (in msec, Bazett's formula):  
  
  
History of Syncope:  

Please Select One
Occurred while on BB
Occurred while off BB
No syncope history
   
  
Genotype:  

Please Select One
LQT1
LQT2
LQT3
Unknown/test negative
   
  
Patient will be continuously treated by beta blockers   
Patient will not be treated by beta blockers  
  
Calculate  
Clear All

  

## Disclaimer

This calculator should NOT be used in

· Patients with a previous history of cardiac arrest

· Patients who are genotype negative or with unknown genotype and has a QTc < 470ms.

· Patients younger than one year and older than 49 years.

This calculator is not intended to replace clinical judgement. The risk of life-threatening arrhythmic events should be interpreted as a continuum in the context of the patient’s clinical condition.
